# Are atrial high rate episodes (AHREs) a precursor to atrial fibrillation?

**DOI:** 10.1007/s00392-019-01545-4

**Published:** 2019-09-14

**Authors:** Ahsan A. Khan, Giuseppe Boriani, Gregory Y. H. Lip

**Affiliations:** 1 Institute of Applied Health Research, University of Birmingham, Birmingham, UK; 2 Liverpool Centre for Cardiovascular Science, University of Liverpool, William Henry Duncan Building, 6 West Derby Street, Liverpool, L7 8TX UK; 3 Division of Cardiology, Department of Biomedical, Metabolic and Neural Sciences, University of Modena and Reggio Emilia, Policlinico Di Modena, Modena, Italy; 4 Aalborg Thrombosis Research Unit, Department of Clinical Medicine, Faculty of Health, Aalborg University, Aalborg, Denmark

**Keywords:** Atrial high rate episodes, Atrial fibrillation, Stroke, Thromboembolism, Anticoagulation

## Abstract

**Abstract:**

Atrial high rate episodes (AHREs), also termed, subclinical atrial tachyarrhythmias or subclinical atrial fibrillation (AF) are an important cardiovascular condition. Advancement in implantable cardiac devices such as pacemakers or internal cardiac defibrillators has enabled the continuous assessment of atrial tachyarrhythmias in patients with an atrial lead. Patients with device-detected AHREs are at an elevated risk of stroke and may have unmet anticoagulation needs. While the benefits of oral anticoagulation for stroke prevention in patients with clinical AF are well recognised, it is not known whether the same risk–benefit ratio exists for anticoagulation therapy in patients with AHREs. The occurrence and significance of AHRE are increasingly acknowledged but these events are still not often acted upon in patients presenting with stroke and TIA. Additionally, patients with AHRE show a significant risk for major adverse cardiovascular events (MACE) including acute heart failure, myocardial infarction, cardiovascular hospitalisation, ventricular tachycardia/fibrillation, which is dependent on AHRE burden. In this review, we present an overview of this relatively new entity, its associated thromboembolic risk and its management implications.

**Graphic abstract:**

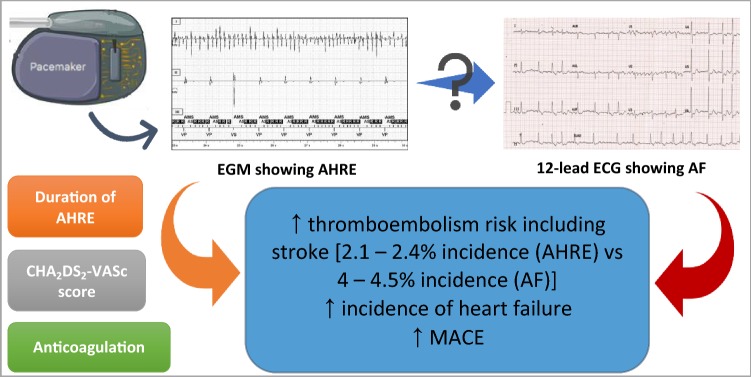

## Introduction

Atrial high rate episodes (AHREs), also termed, subclinical atrial tachyarrhythmias or subclinical atrial fibrillation (AF) are an important cardiovascular condition. AHRE is defined as an episode of fast heart rate, i.e. more than 180 beats per minute (bpm) lasting at least 5 min as per the European Society of Cardiology (ESC) 2016 guidelines [1]. Cardiovascular implantable electronic devices (CIEDs), such as permanent pacemakers (PPM), implantable cardioverter defibrillators (ICDs), cardiac resynchronisation therapy (CRT) devices [pacemakers (CRT-P) and defibrillators (CRT-D)], are being used increasingly throughout the world. Such devices with an atrial lead can detect episodes of atrial arrhythmias, regardless of the presence of symptoms. This relatively new entity is distinct from detection of AF via portable ECG monitoring and new devices and applications capable of recording ECG such as Apple Watch, which requires consideration on its own merit.

Patients with CIEDs represent a unique population with multiple comorbidities predisposing to atrial arrhythmias. Most of these AHREs are asymptomatic and sensed in asymptomatic individuals at a routine pacing clinic follow-up. A recent study showed that more than half of patients with ICD for primary prophylaxis with sinus rhythm at baseline developed new AF or ventricular arrhythmia after 6 years [2]. The onset of these arrhythmias was found to be independent prognostic factors for increased mortality in this group of patients [2].

The incidence of AHRE in patients without a history of AF is approximately 25% after 1 year and 35% after 2 years of follow-up [3–5]. AHRE differ from AF in the manner of documentation, i.e. AF is established on an electrocardiogram (ECG), whereas AHREs are solely recorded on a CIED read-out [1]. Several factors have been identified to be associated with AHRE onset. These include, age, prior AF, white cell count and high C-reactive protein (CRP) [6]. Furthermore, similarities are being drawn between paroxysmal AF (PAF) and AHREs. PAF, as opposed to permanent AF, is transient, infrequent and often asymptomatic. There is evidence to suggest that asymptomatic PAF episodes occur with much greater frequency than symptomatic PAF [7]. Thus, patients with CIEDs present a unique opportunity to screen for and unmask silent AF episodes.

Irrespective of AF diagnosis, patients with device-detected AHREs are at an elevated risk of stroke and may have unmet anticoagulation needs [8]. The incidence of AHRE detected by CIED can reach 50% but less than 25% of these patients are treated with an oral anticoagulant [9]. While the benefits of oral anticoagulation for stroke prevention in patients with clinical AF are well recognised, it is not known whether the same risk–benefit ratio exists for anticoagulation therapy in patients with AHREs [8]. Some studies do suggest that AHRE and AF differ regarding thromboembolism risk. In AHRE patients, the thromboembolic risk appears to be lower than in clinical AF [4, 10–13].

The pathophysiology behind increased thromboembolic risk in patients with AHRE has not yet been clearly established. Atrial abnormalities that predispose to development of atrial arrhythmias increase the risk of thromboembolism, irrespective of the presence of the arrhythmia [14]. This hypothesis is supported by the notion that the majority of strokes in patients with CIED-detected AHRE do not occur within 30 days of the last episode of AHRE [15, 16]. Thus, a relation between severity of atrial cardiomyopathy and AHRE burden is yet to be recognised.

## Are AHREs a precursor to atrial fibrillation?

Advances in implantable cardiac devices, such as PPM and ICD, allow long-term continuous heart rhythm monitoring and have enabled the continuous assessment of atrial tachyarrhythmias in patients with an atrial lead [17, 18]. However, each CIED manufacturer uses a different definition for AHRE which makes it difficult to standardise the captured data. There is a tendency in clinical practice to assume that AHREs detected by CIEDs are equivalent to having AF. The CIEDs’ AHRE algorithm has a high sensitivity for detection of AF, ranging from 94 to 100% [17, 19, 20]. Nonetheless, utilisation of device-detected AHREs to diagnose AF is not perfect due to false negatives, especially when the duration of AHRE is brief, i.e. less than 30 s [21].

The researchers working on the Asymptomatic Atrial Fibrillation and Stroke Evaluation in Pacemaker Patients and the Atrial Fibrillation Reduction Atrial Pacing Trial (ASSERT) independently reviewed nearly 6000 AHREs (defined in their study as episodes of heart rate more than 190 beats per minute for more than 6 min) and discovered that 17.3% were false positives [22]. However, the rate of false positives was reduced to 3.3% when using a longer threshold of 6 h for AHRE duration suggesting that longer the duration of AHRE, the lower the number of false-positive detections [22]. Possible reasons for false positives include oversensing of the atrial lead, runs of pacemaker lead-mediated arrhythmia, premature atrial complexes, far-field R-wave sensing, or other external interference.

Irrespective of the above, the occurrence and significance of AHRE are increasingly acknowledged but these events are still not often acted upon in patients presenting with stroke and TIA. An analysis of the Atrial Fibrillation Follow-up Investigation of Rhythm Management (AFFIRM) study discovered that 12% of patients were asymptomatic at baseline [23]. These patients had a lower incidence of serious heart disease but more strokes [23]. 5% of patients presenting with an acute stroke have previously undetected AF on admission. Subsequent intermittent 12 lead ECG or Holter monitoring has identified higher incidences of undiagnosed AF in stroke survivors [24, 25]. The early detection of AHREs is thus clinically relevant considering that they have been shown to be associated with an elevated risk of thromboembolic events and death, such as 1.8%/year in the IMPACT trial, a multicentre randomised study of anticoagulant guided by remote rhythm monitoring in patients with implantable cardioverter–defibrillator and CRT-D devices [4, 8, 15, 26–28].

Most of the data on AHRE have been obtained from patients with cardiac devices in situ. Many of these patients have sinus node disease and/or ventricular pacing which are associated with a higher incidence of AF. Thus, the prevalence of AHRE may be lower in the general population [29, 30]. A growing body of clinical data support the hypothesis that AHREs are associated with an elevated risk of stroke [8]. A recent meta-analysis by Uittenbogaart et al. showed that patients with an AHRE burden over 6 min had an increased risk of thromboembolic event when compared with patients without AHRE but this risk did not increase for an AHRE burden over 6 h (hazard ratio (HR) 1.82 vs 1.78) [31]. In a second meta-analysis, they discovered that only patients with AHRE burden over 24 h had an increased risk for stroke (HR 3.2, 95% confidence interval (CI) 1.75–5.86) while patients with an AHRE burden < 24 h did not [31].

Benezet–Mazuecos et al. prospectively analysed the incidence of AHRE (defined as heart rate ≥ 225 bpm and lasting > 5 min) in 109 patients and the presence of silent ischaemic brain lesion on computed tomography (CT) scan [32]. Multivariate analysis demonstrated that AHRE was an independent predictor of silent ischaemic stroke (HR 9.76, 95% CI 1.76–54.07; *p* < 0.05) [32].

An ancillary study of the Mode Selection Trial (MOST) discovered that patients (*n* = 312) with sinus node dysfunction who experienced AHRE (defined as atrial rate > 220 bpm for 10 consecutive beats) were more likely to have adverse clinical outcomes, including 6 times as likely to develop AF and twice as likely to have a stroke or die than patients without AHRE (20.6% vs 10.5%; HR 2.79, 95% CI 1.51–5.15, *p* = 0.001; no annual rates reported) [19]. The study was, however, limited by its retrospective design, small sample size and that 80% of enrolled patients had a previous history of supraventricular arrhythmia.

The TRENDS trial (A Prospective Study of the Clinical Significance of Atrial Arrhythmias Detected by Implanted Device Diagnostics) was a prospective, multicentre observational study looking at 2486 patients with a CIED and CHADS_2_ [congestive heart failure, hypertension, age ≥ 75 years, diabetes mellitus and prior stroke (doubled)] score ≥ 1 showed that the annual thromboembolic risk doubled in patients with a high atrial tachycardia (AT)/AF burden (defined as AHRE of ≥ 5.5 h), compared to patients with zero or low burden, i.e. < 5.5 h per day (2.4% vs 1.1% per year; HR 2.20, 95% CI 0.96–5.05, *p* = 0.06) [10]. The AHRE itself was defined as > 175 bpm lasting at least 20 s. The risk remained elevated even after adjustment for other risk factors. This study did include patients with prior history of AF, although incidental AHRE was noted in 45% of 1988 patients without a documented history of prior AF. Similarly, Turakhia et al. showed that patients who suffered a stroke had more often AHRE lasting ≥ 5.5 h in the 30 days preceding the stroke compared with a control period of days 91 to 120 prior to the stroke in the same patients (HR 4.2, 95% CI 1.5–13.4) [33].

More recently, the Asymptomatic Atrial Fibrillation and Stroke Evaluation in Pacemaker Patients and the Atrial Fibrillation Reduction Atrial Pacing Trial (ASSERT) was designed as a prospective, multicentre, observational study to assess if AHREs can be associated with an increased risk of ischaemic stroke in patients with no prior history of AF [4]. The study followed 2580 patients with recently implanted pacemaker or ICD for a mean of 2.5 years and found that the presence of AHRE (defined as AHRE > 190 bpm lasting > 6 min) was predictive of stroke or systemic embolism even after adjustment for predictors of stroke (HR 2.50; 95% CI 1.28–4.89; *p* = 0.008) [4].

The subanalysis of the ASSERT study looking at the duration of AHRE and thromboembolic risk using time-dependent Cox regression model showed that thromboembolic risk only increased in patients with AHRE lasting more than 24 h (HR 3.24, 95% CI 1.51–6.95, *p* = 0.003) compared to patients without AHRE [34]. For AHRE lasting < 24 h, the thromboembolic risk appeared to be similar to patients without AHRE [34]. Furthermore, the number of AHRE did not affect thromboembolic risk [4]. Interestingly, the annualised thromboembolic event rate was found to be equal to 2.1% in the subgroup with CHADS_2_ score > 2, which was similar to TRENDS (2.4%). This is, however, still below the 4–4.5% annual rate expected in clinical AF patients with a similar risk profile [35].

Data from TRENDS and ASSERT are further supported by multiple smaller prospective trials which assessed the relationship between AHREs and thromboembolic events in patients with CIEDs. Capucci et al. looked at 725 patients with dual chamber PPM and discovered that AHRE lasting < 24 h did not significantly increase embolic risk, while episodes > 24 h did (odds ratio 3.1) [36]. Botto et al. in a separate study looked at 562 patients with dual-chamber PPM and followed them for 1 year post-implantation [37]. They stratified patients using a combination of AHRE burden and CHADS_2_ score [37]. They discovered that separate populations with different stroke risk emerged [37]. Patients with AHRE > 5 min and CHADS_2_ score of ≥ 2 and cumulative AHRE > 24 h with CHADS_2_ score > 1 had an annualised thromboembolic event rate of as high as 5% [37].

There are several other studies that have established a clear association between AHRE and increased risk of stroke as summarised in Table 1 [29, 30, 38, 39]. However, apart from the ASSERT trial, all other studies have included patients with a history of AF. Hence, the sole effect of AHRE on thromboembolic event cannot be reliably assessed. Additionally, half of these trials have included small sample sizes. Furthermore, there have been differences among the trials in the characteristics of the patient populations, use of anticoagulation, the pacing algorithm used and the atrial lead positions which will lead to statistical limitations and the inferences from these studies. Low frequency of thromboembolic events leaves an extensive room for statistical error associated with the estimation of thromboembolic event rates or risk related to predictors of such events.

**Table 1:** Overview of AHRE trials regarding thromboembolism risk

Trial	*n*	Prior AF (%)	Mean CHADS_2_ score	Definition of AHRE		AHRE + annual TE (%)	AHRE − annual TE (%)	RR for TE	*p*
				atrial rate	Duration				
ASSERT [4]	2580	0	2.2	> 190 bpm	> 6 min	1.7	0.7	2.5	0.007
AT500 [36]	725	100	–	AT/AF	> 24 h	–	–	3.1	0.044
Benezet-Mazuecos [32]	109	31	2.3	≥ 225 bpm	≥ 5 min	–	–	3.04	< 0.05
ClinicalService [27]	3907	21	2	-	-	0.21	–	-	–
IMPACT [15]	227	17.6	2.5	≥ 220 bpm	≥ 5 min	3.1	2.3	− 35.3	0.251
Kawakami et al. [48]	343	24	2.3	≥ 175 bpm	> 6 min	8.48	2.8	2.87	0.03
Li et al. [49]	594	0	3.2	≥ 175 bpm	≥ 5 min	1.85	1.14	1.31	0.582
Miyazawa et al. [50]	856	24.8	1.9	≥ 175 bpm	≥ 5 min	2.6	0.9	3.4^a^	0.01
MOST [19]	312	60	–	> 220 bpm	> 5 min	–	–	2.8^a^	0.001
PANORAMA [27]	3556	25	2	> 175 bpm	≥ 20 s	0.28	–	–	–
SOS AF Project [27]	10,016	24	2	> 175 bpm	≥ 20 s	1.28	0.72	2.05	0.005
TRENDS [10]	2486	20	2.2	> 175 bpm	≥ 5.5 h	2.4	1.1	2.2	0.06
Turakhia et al. [33]	9850	41	3.2	AT/AF	≥ 5.5 h	–	–	4.2	< 0.05

*AT* atrial tachycardia, *bpm* beats per minute, *RR* relative risk; *TE* thromboembolism, *h* hour(s), *min* minute(s), *s* second(s)

^a^Combined endpoint of death and non-fatal stroke

Studies have included patients with various devices implanted, each with their own detection algorithm, which could translate to differences. AHREs were predominantly confirmed by device algorithms with very high sensitivity (100%), specificity (97%) and positive predictive value (100%) [36]. This together with the long duration of AHREs detected (usually in hours) and included in most studies such as ASSERT suggest that chances of missing or detection of false-positive AHRE were remote. Consequently, information on false-positive AHREs identified in these various studies has not been provided by the researchers. Furthermore, additional steps to confirm AHRE by reviewing of intracardiac electrograms were limited to a few studies. In some studies, this was performed by cardiac electrophysiology experts whereas in others it was done by clinicians with no specification of their expertise levels.

Although an increased risk of thromboembolism and AHRE has been shown in multiple studies, a clear temporal relationship between AHRE and subsequent stroke risk has not yet been identified. In a subgroup analysis of TRENDS, approximately 50% of patients who suffered a stroke or TIA had an AHRE episode recorded prior to the event, 25% of stroke patients had an AHRE within a month of the event and only 15% were associated with an AHRE during the event [16]. These findings are similar to ASSERT data where only half of the patients sustained an AHRE prior to their thromboembolic stroke event, 12% within 30 days and 2% during the stroke event [40]. It is plausible that instead of causing embolic events, AHREs are simply a marker for thromboembolic risk [40]. Besides, studies that had patients with prior AF included or considered required recent presence of AF or two AF episodes documented on a 12 lead ECG [36]. Thus, the AF burden in these patients would be higher as oppose to patients with no previous history of AF.

Of note, the risk due to AHRE may extend beyond increased risk of stroke. A study looking at 224 patients with no history of AF who underwent dual-chamber PPM discovered that AHREs were associated with a significant increase in cardiovascular mortality (HR 2.80; 95% CI 1.24–6.31; *p* = 0.013) and stroke mortality (HR 1.79, 95% CI 0.98–3.26; p = 0.059) [41]. A recent study by Pastori et al. showed that patients with AHRE show a significant risk for major adverse cardiovascular events (MACE) including acute heart failure, myocardial infarction, cardiovascular hospitalisation, ventricular tachycardia/fibrillation, which is dependent on AHRE burden [28].

## Management of AHRE

Anticoagulation therapy is well established for the management of stroke prophylaxis in patients with AF diagnosed by standard ECG. At present, the management of patients with device-detected AHRE remains controversial with uncertainties surrounding false positives, duration of the longest AHRE episode, the cumulative duration and the individual stroke risk [9]. There is at present limited evidence from randomised clinical studies to inform management of subclinical AF detected by AHRE interrogation through cardiac devices. Thus, selecting the most appropriate anti-thrombotic therapy for patients with AHRE is one of the evidence gaps highlighted by the ESC guidelines on the management of AF [1].

Recently, 46 European device-implanting centres took part in a European Heart Rhythm Association survey to capture the current clinical practice [42]. 53% of cardiologists recommended anticoagulation when CHA_2_DS_2_-VASc (congestive heart failure, hypertension, age ≥ 75 years (doubled), diabetes mellitus, prior stroke (doubled), vascular disease, age ≥ 65 years and sex) score was 2–3 as opposed to 70% when CHA_2_DS_2_-VASc score was 4 when presented with a clinical scenario where a single AHRE was detected lasting more than 6 min [42]. This accurately represents the heterogeneity in the clinical attitude towards management of AHRE [42]. Overall, the inclination was shown towards favouring anticoagulation in those patients with a higher CHA_2_DS_2_-VASc score, multiple AHRE and longer duration of episodes [42].

Taking into account the current literature and while awaiting outcome from ongoing randomised clinical trials comparing oral anticoagulation vs no anticoagulation in patients with device-detected AHRE such as ARTESiA (Apixaban vs Aspirin; *n* = 4000) and NOAH (Edoxaban vs Aspirin; *n* = 3400) with a primary endpoint composite of stroke, systemic embolism and cardiovascular death, the European Society of Cardiology has provided some recommendations with regard to the management of AHRE patients [1, 43, 44].

**Fig. 1:**
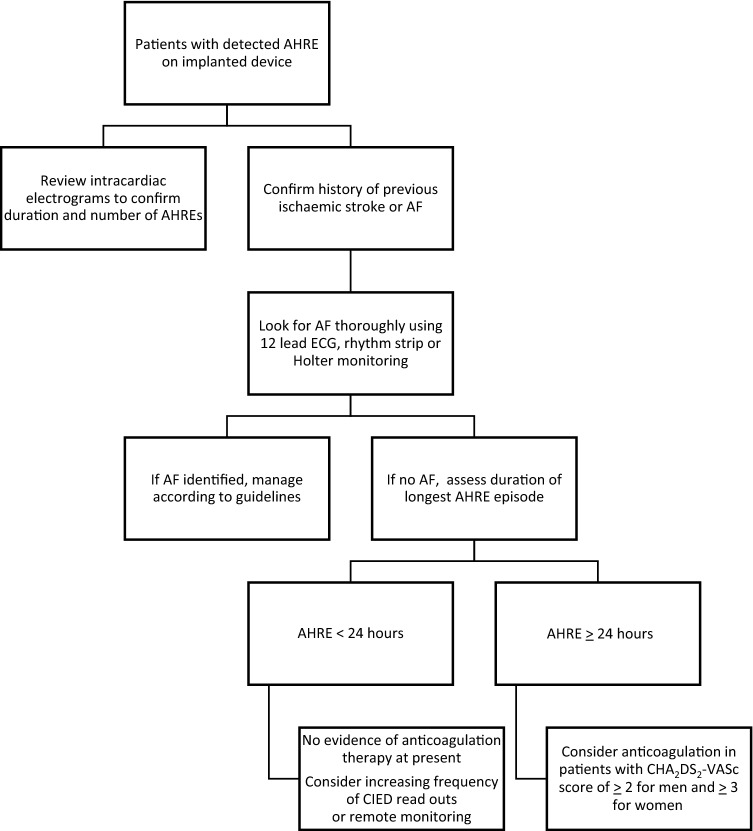
Proposed flowchart for management of patients with device-detected AHRE [1]

The ESC 2016 guidelines recommend that in patients with detectable AHRE on implanted device should be assessed for history of previous ischaemic stroke or episode of AF [1]. If there is no evidence of such, AF should be thoroughly looked for using 12-lead ECG, rhythm strip or prolonged review of intracardiac electrograms [1]. If still no clinical AF, then patients with AHRE ≥ 24 h (high burden) and CHA_2_DS_2_-VASc score of ≥ 2 for men and ≥ 3 for women should be anticoagulated [1]. Figure 1 represents a flowchart to summarise this recommendation.

## Conclusion

AHREs are a common finding in patients with cardiac implanted devices without a history of AF. Following advances in cardiac monitoring, it is likely that AHRE will be increasingly reported in the future. AHREs are often considered equivalent to clinical paroxysmal AF. This view has been supported by previous studies demonstrating that AHREs have a high correlation with clinically documented AF [45].

It is now increasingly recognised that AHRE are associated with an increased thromboembolic risk, although lower than in clinical AF, dependent on the duration of the AHRE. Thus, the initiation of anticoagulation therapy is naturally tempting. The lack of concrete evidence for a temporal relationship between AHRE and stroke, lack of specific recommendations regarding use of CIEDs for diagnosis and management of AF, sparse evidence on the critical threshold for duration/number of AHRE burden and absence of studies demonstrating benefit of oral anticoagulation therapy in patients with device-detected AHRE reflects the variation in individual clinical practice.

The pathological and prognostic significance of AHRE has not been completely established. Despite the overall stroke rate in patients with AHREs appears to be less than that found with clinical AF, it is still imperative to identify a certain high-risk population who deserve anticoagulation therapy, provided that embolic risk exceeds the risk of serious bleeding. Thus by combining AHRE burden with CHA_2_DS_2_-VASc score and HAS-BLED score, one can individualise oral anticoagulation therapy for appropriate patients at high risk of stroke [37]. Based on limited available evidence, it appears reasonable to commence anticoagulation in patients without AF and at least one episode of AHRE lasting ≥ 24 h and a CHA_2_DS_2_-VASc score of ≥ 2 for men and ≥ 3 for women as per the ESC 2016 guidelines, while awaiting definite answers from ongoing randomised clinical trials, ARTESiA and NOAH. Patients with shorter AHRE (AHRE < 24 h) should at present be frequently monitored with remote monitoring for propagation to high burden AHRE and/or development of AF until more definitive evidence comes to light [46].

There is an unmet need for high quality evidence. Future studies must consider a standardised definition of AHRE, as past studies have used various definitions. They should also consider how CIEDs algorithms operate and detect AHREs as a recent study found significant variation in diagnostic accuracy among devices and according to the level of operator expertise [47]. The use of oral anticoagulation therapy for stroke prophylaxis in patients with AHRE should also be evaluated including cost effectiveness. Future studies should also address the impact of treatment on patient’s quality of life. Such studies will improve the existing variable and poorly informed evidence and help inform shared decision-making, clinical guideline development and health policy.
